# Controlled Release of 5-Aminosalicylic Acid (5-ASA) from New Biodegradable Polyurethanes

**DOI:** 10.3390/molecules15042257

**Published:** 2010-03-30

**Authors:** El-Refaie Kenawy, Salem S. Al-Deyab, Mohamed H. El-Newehy

**Affiliations:** 1 Petrochemical Research Chair, Chemistry Department, College of Science, King Saud University, P.O. Box: 2455 Riyadh 11451, Saudi Arabia; 2 Polymer Research Group, Chemistry Department, Faculty of Science, Tanta University, Tanta 31527, Egypt

**Keywords:** 5-aminosalicylic acid, azo polymers, biodegradable polymers, polyurethane, drug delivery systems

## Abstract

Segmented polyurethanes containing azo aromatic groups in the main chain were synthesized by reaction of 3,3′-azobis(6-hydroxybenzoic acid) (ABHB), 5-[4-(hydroxyphenyl)azo] salicylic acid (HPAS), and 5-[1-hydroxynaphthyl)azo] salicylic acid (HNAS) with hexamethylenediisocyanate (HDI). All synthesized monomers and polymers were characterized by elemental analysis, FTIR, ^1^H-NMR spectra, TGA and DSC analysis. All the synthesized azo polymers showed good thermal stability and the onset decomposition temperature of all these polymers was found to be above 195 °C under nitrogen atmosphere. The release of 5-ASA under physiological conditions (pH = 7.8 and pH = 1.5) was investigated at body temperature (37 °C). The release rate of 5-ASA increased with increasing pH (*i.e.*, 7.8 > 1.5).

## 1. Introduction

The colon has recently received great attention as a potential site for the delivery of pharmaceutical moieties. The factor usually exploited for this is the pH gradient between the stomach and the colon [1,2,3,4,5,6,7]. Colon-specific drug delivery has been developed for treating ulcerative bowel disease, colitis, colon cancer and infectious diseases where it is necessary to attain a high concentration level of active agent in the large intestine [8,9]. Delivery of drugs via the colon offers numerous therapeutic advantages. The successful delivery of drugs to the colon via the gastrointestinal (GI) tract requires the protection of a drug from being released in the stomach and small intestine. This can be achieved by the use of a special drug delivery system that can protect the drug during its transfer to the colon [10,11,12].

Several approaches have been developed for targeted colon drug delivery. Colon-specific devices may be classified into pH-dependent systems, positioned-release systems, low molecular weight prodrugs, macromolecular prodrugs, and biodegradable polymers. Biodegradable polymers, including azo polymers, are known to break down selectively within the colon [1]. Azo polymers comprise a class of both aliphatic and aromatic polymers containing the azo functionality. Aliphatic azo polymers are thermally unstable; on the other hand, aromatic azo polymers are heat resistant due to stabilization by resonance [13].

Colon targeting has become the focus of increasing interest, and various delivery systems have been developed. On the basis of synthetic methodology and the resulting delivery forms, colon targeting systems can be classified into hydrogels, coating systems, and polymeric prodrugs [13]. The colon-targeting materials including synthetic polymers containing aromatic azo groups are known to be metabolized by the intestinal bacteria [8]. Reduction of the azo-bond to hydrazo compounds and/or amines is believed to be responsible for the degradation phenomena [1,14,15,16,17]. The generation of the active moiety, 5-aminosalicylic acid (5-ASA), by the action of the intestinal microflora has been the key for colon-specific drug delivery to the large intestine based upon the enzymatic reduction of the azo bond [13].

In this study, segmented polyurethanes containing azo aromatic groups in the main chain were synthesized by reaction of 3,3′-azobis(6-hydroxybenzoic acid) (ABHB), 5-[4-(hydroxyphenyl)azo] salicylic acid (HPAS), 5-[1-hydroxynaphthyl)azo] salicylic acid (HNAS), and 5-[4-(hydroxyphenyl) azo] isopropyl salicylate (HPAIS) with hexamethylenediisocyanate (HDI). The resulting biodegradable polyurethanes containing 5-aminosalicylic acid (5-ASA) in the main chain were used as polymeric prodrug systems. The use of these prodrug systems can protect 5-ASA during its transfer to the colon. The release of 5-ASA was investigated under physiological conditions (pH = 7.8 and pH = 1.5) at body temperature (37 °C).

## 2. Results and Discussion

The colon has recently received great attention as a potential site for the delivery of drugs. The factor usually exploited for this is the pH gradient between the stomach and the colon [1]. In this paper, a new system of biodegradable polymers including azo polymers was synthesized to optimize the therapeutic properties of the drugs and render them safer and more effective.

### 2.1. Monomers synthesis

**Scheme 1:**
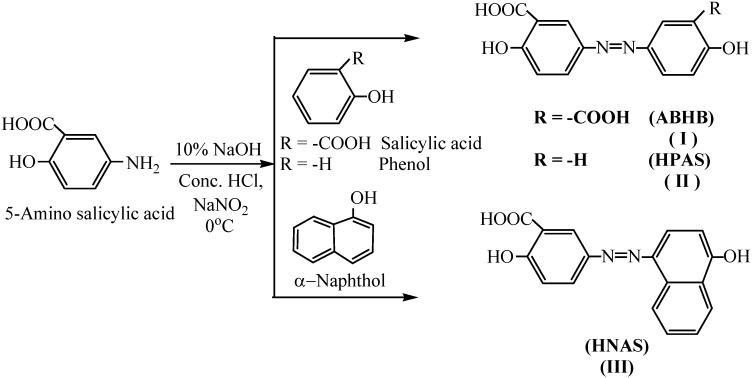
Synthesis of different 4,4′-dihydroxyazobenzene derivatives **I**–**III**.

Monomers **I**-**III**, as azo dyes, were prepared by diazotization of 5-aminosalicylic acid using sodium nitrite in the presence of hydrochloric acid. Subsequent coupling with salicylic acid, phenol or α-naphthol produced monomers **I, II** and **III**, respectively, in good yield (Scheme 1). The monomers purity was analyzed by thin-layer chromatography. The structure of the prepared monomers was confirmed by elemental analysis, FTIR, and ^1^H-NMR. The elemental microanalyses, as shown in Table 1, were in agreement with the calculated values.

### 2.2. Synthesis of Azo-Containing Polyurethane

**Scheme 2:**
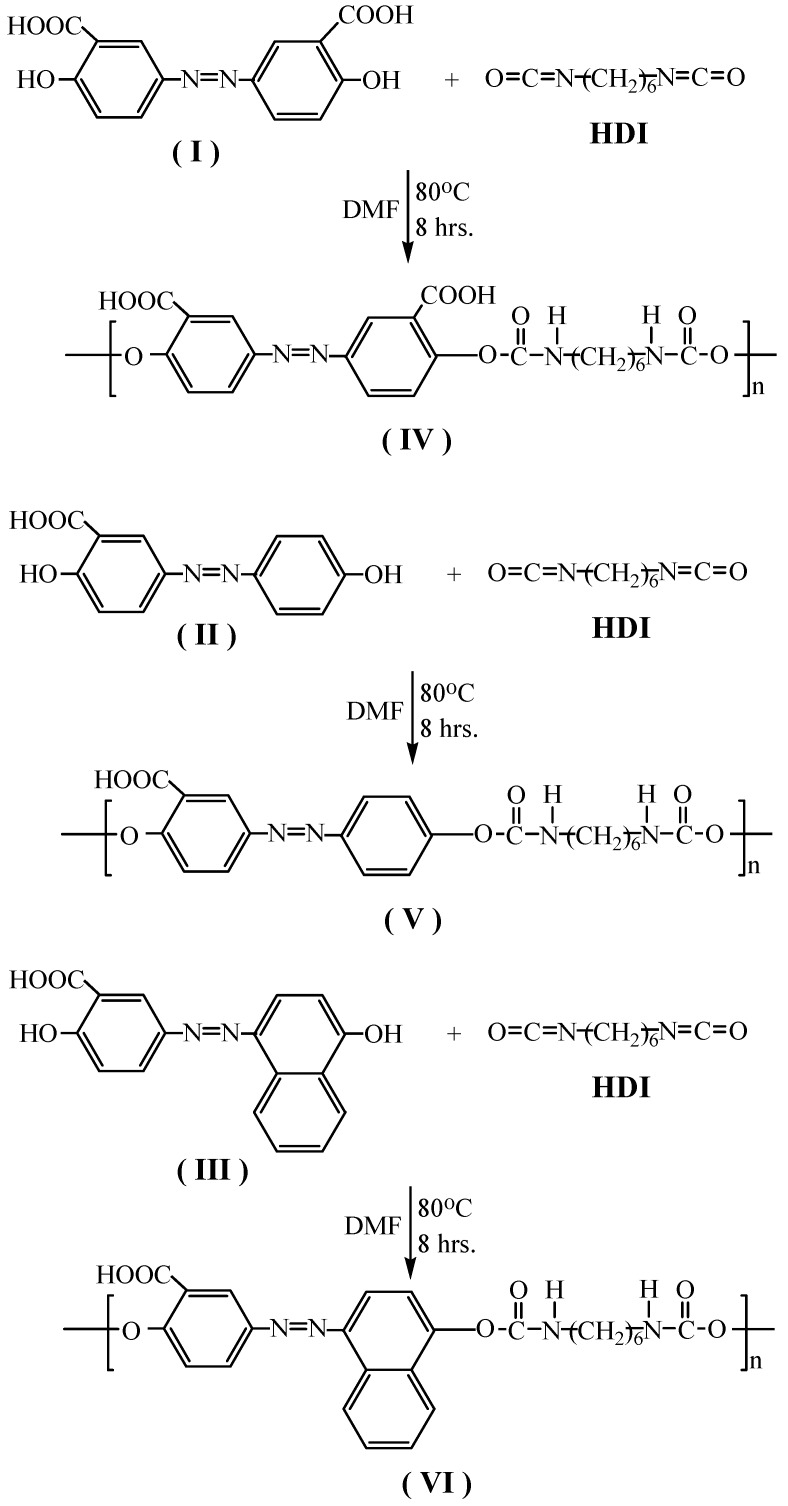
Polymer synthesis.

The azo-containing polyurethanes were prepared by polycondensation of 1,6-hexamethylene diisocyanate (HDI) with the different *p*-aminosalicylic acid based monomers **I**–**III** in 1:1 ratio as shown in Scheme 2. The polymers were precipitated in good yields (75–92%) and were characterized by spectroscopic methods and elemental microanalysis, which as shown in Table 1, were in agreement with the calculated values.

### 2.3. Thermal Analysis

The azo polymers were degraded in non-oxidative degradation steps depending on the structure of the polyurethanes. Generally, the thermograms showed that the onset decomposition temperature (*T_on_*) of all these polymers was above 195 °C under nitrogen; this indicates the good thermal stability of the synthesized azo polymers.

It was found that polymer **IV** was degraded in five steps. The first step was a weight loss of about 1.3% at the temperature range of 25–200 °C, which represents the evolution of the moisture. The second step was a weight loss of about 24.6% at the temperature range of 200–340 °C, which represents the start of the decomposition of polymer **IV**. The third step was a weight loss of about 27.8% at the temperature range of 340–445 °C, the fourth step was a weight loss of about 19.81% at the temperature range of 445–513 °C, and the last step was a weight loss of 21.8% at the temperature range of 513–625 °C, which represent the main thermal degradation of polymer **IV** and the carbonization of the products to ash, which represents about 4.7% at 800 °C.

For polymer **V**, it was found that it was degraded in four steps. The first step was a weight loss of about 0.9% at the temperature range of 25–200 °C, which represents the evolution of the moisture. The second step with a weight loss of about 22.1% at the temperature range of 200–337 °C, which represents the initial decomposition of polymer **V**. The third step with a weight loss of about 22.2% at the temperature range of 337–453 °C. The last step as a weight loss of 48.3% at the temperature range of 453–609 °C, which represents the main thermal degradation of polymer **V** and the carbonization of the products to ash, which represented about 6.6% at 800 °C.

Polymer **VI** was found to be degraded in three steps. The first step was a weight loss of about 1.8% at the temperature range of 25–195 °C, which represents the evolution of the moisture. The second step was a weight loss of about 55.6% at the temperature range of 195–422 °C, which represents the main thermal degradation of polymer **VI**. The last step was a weight loss of 20.9% at the temperature range of 422–532 °C, which represent the carbonization of the products to ash. The residue at 800 °C was found to be 21.8%.

### 2.4. Determination of the Total 5-ASA Content

To determine the total content of the polymers derived from 5-ASA, it was necessary to carry out a fast hydrolysis of the drug from the polymers. Heating the polymeric systems at 60 °C in phosphate buffer with pH 7.8 hydrolyzed the drug-polymer bond. The fast hydrolysis of 5-ASA in buffer solution led to total 5-ASA contents that were found to be 186.93, 143.74, and 125.82 (mg of 5-ASA)/(g of polymer) for polymers **IV**, **V**, and **VI**, respectively. The results obtained by the alkaline hydrolysis were in agreement with the calculated values and those obtained from elemental analysis.

### 2.5. In Vitro Drug Release

**Figure 1:**
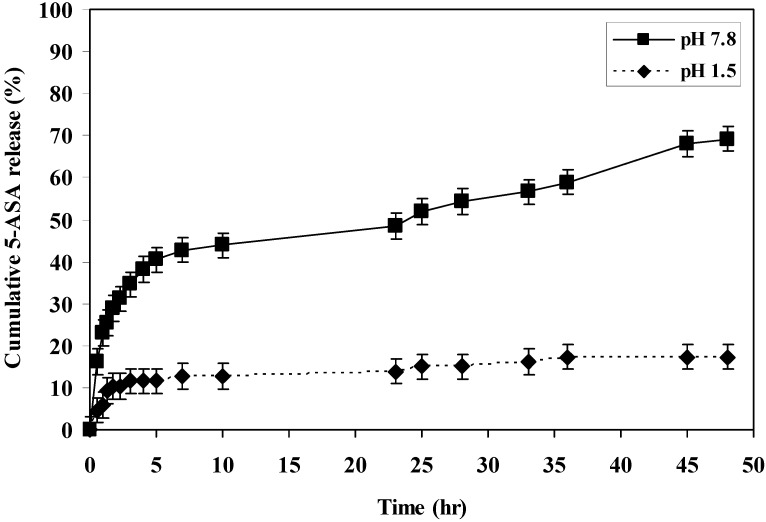
*In vitro* 5-ASA release from polymer **IV** in phosphate buffer solutions (pH 7.8 and 1.5) at 37 °C.

**Figure 2:**
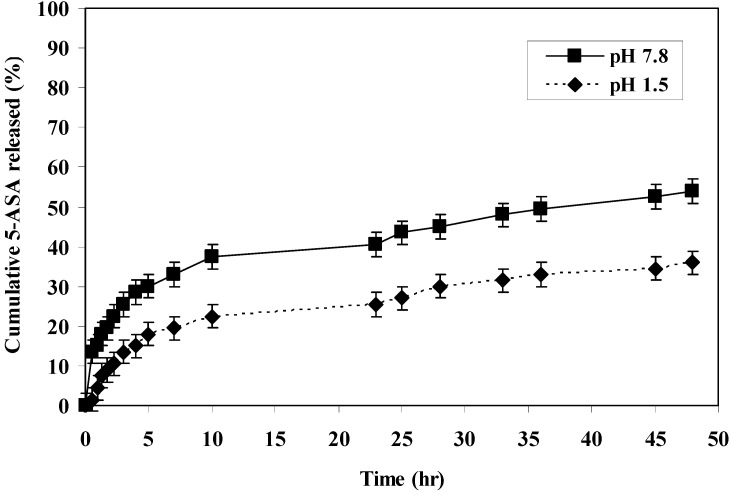
*In vitro* 5-ASA release from polymer **V** in phosphate buffer solutions (pH 7.8 and 1.5) at 37 °C.

**Figure 3:**
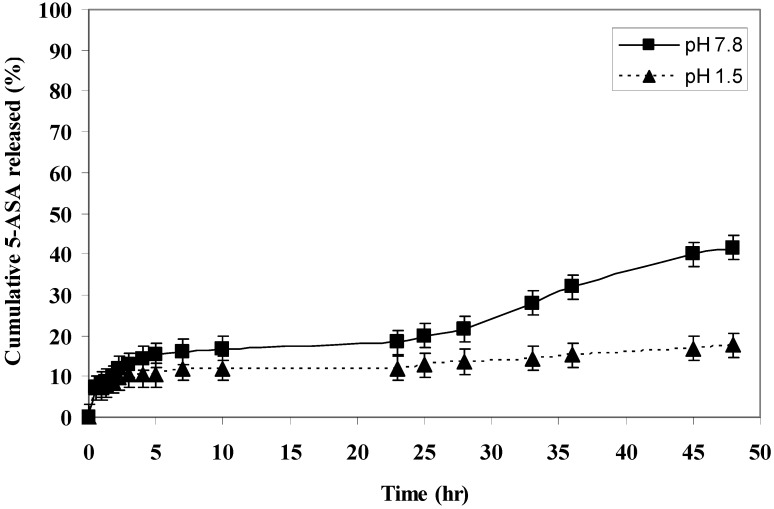
*In vitro* 5-ASA release from polymer **VI** in phosphate buffer solutions (pH 7.8 and 1.5) at 37 °C.

**Figure 4:**
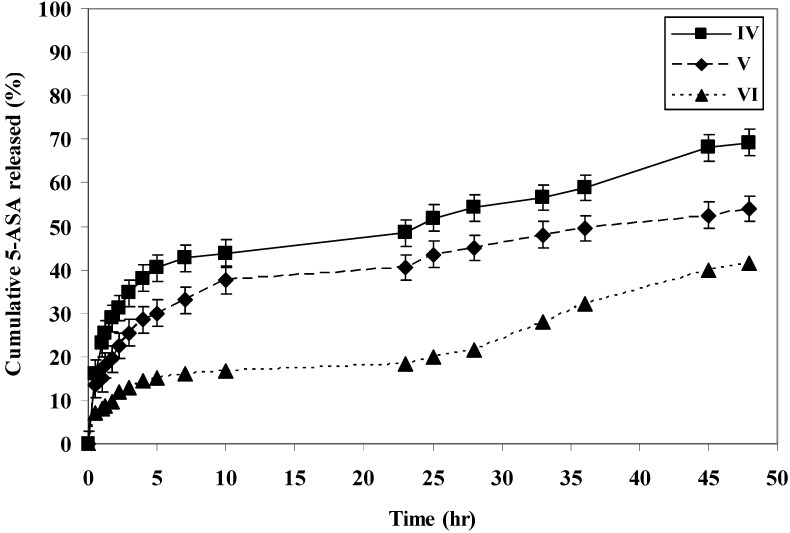
*In vitro* 5-ASA release from polymers IV-VI in phosphate buffer solution (pH 7.8) at 37 °C.

**Figure 5:**
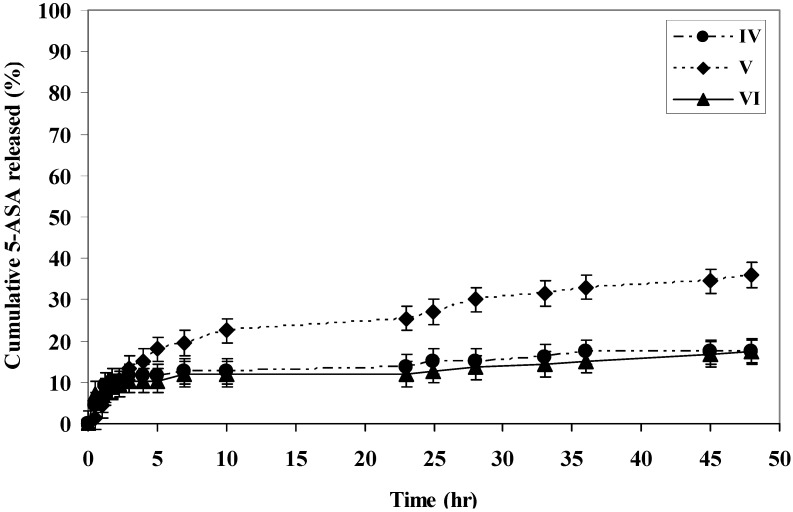
*In vitro* 5-ASA release from polymers IV–VI in phosphate buffer solution (pH 1.5) at 37 °C.

The rate of 5-ASA released from the polymers was measured at pH 7.8 and 1.5 at 37 °C. The rate of release showed dependence on the pH of the medium and on the polymer microstructure. Generally, it was found that the rate of release of 5-ASA increased as the pH increased in alkaline medium (*i.e.,* within the colon pH) as shown in (Figure 1, Figure 2, Figure 3, Figure 4 and Figure 5).

At pH 7.8, polymer **IV**, polymer **V** and polymer **VI** released 69%, 54% and 41% of 5-ASA, respectively, after two days at 37 °C. At pH 1.5 at 37 °C, the total amounts of 5-ASA released after two days were found to be 17%, 36%, and 17% for polymer **IV**, **V** and **VI**, respectively. At pH 1.5 and 37 °C temperature, the total amounts of 5-ASA released after two hours were found to be around 10%, respectively. This means that more than 90% of the drug will pass to the colon without hydrolysis. Therefore, this indicated that the system will be useful for colon drug targeting. Generally, as the number of carboxylic acid groups on the monomeric units along the polymer chain increased, the hydrophilicity of the polymer increased, and the rate of hydrolysis increased, and consuquently, the amount of 5-ASA released increased.

## 3. Experimental

### 3.1. Materials

5-Aminosalicylic acid (5-ASA), salicylic acid, phenol, α-naphthol, sodium nitrite, and 1,6-hexamethylenediisocyanate (HDI) were purchased from Aldrich and were used as received. *N*,*N*- dimethylformamide (DMF) and methanol were received from El-Nasr for Chemicals Company, Cairo, Egypt and were dried and distilled before use.

### 3.2. Characterization Techniques

Elemental microanalyses were recorded on a Heraeus instrument. ^1^H-NMR Spectra were recorded on a JEOL JNM-PM X 90 Si-NMR instrument. IR spectra were recorded from KBr pellets on a Perkin-Elmer 1430 Ratio Recording Infrared Spectrophotometer. Thin-layer chromatography (TLC) was carried out with silica-gel-precoated plastic sheets containing a multifuorescent indicator (UV-254/366 NM) and supplied by San Gabriel (San Gabriel, CA, USA). Thermogravimetric analysis (TGA) was performed with a TGA-50 Shimadzu-Japan. Samples of 5–6 mg were heated in the temperature range 25–800 °C at a scanning rate of 20 °C/min under nitrogen atmosphere with a flow rate of 20 mL/min.

### 3.3. Preparation of Phosphate Buffer (PB) Solution

Phosphate buffer solution (PB) was prepared as previously described as following [7]: a pH 7.8 buffer solution was prepared by dissolving sodium phosphate dibasic (21.7 g) and potassium phosphate monobasic (2.6 g) in deionized water (1 L) and the pH was adjusted to 7.8 using 0.1 N sodium hydroxide. A pH 1.0 buffer solution was prepared by dissolving sodium phosphate dibasic (21.7 g) and potassium phosphate monobasic (2.6 g) in deionized water (1 L) and pH was adjusted to 1.5 using 0.1 N hydrochloric acid.

### 3.4. Monomer Synthesis

#### 3.4.1. Synthesis of 3, 3′-Azobis(6-hydroxybenzoic acid) (ABHB, **I**)

In 500 mL conical flask, 5-aminosalicylic acid (1.53 g, 10 mmol) was dissolved in a mixture of concentrated hydrochloric acid (10 mL), water (25 mL) and ice (25 g). The solution was cooled with stirring in an ice bath to 0 °C until it become clear, then a solution of sodium nitrite (3.45 g, 50 mmol) in water (7.5 mL) was added dropwise during 10 min, and the reaction mixture was further stirred for 20 min in an ice bath at 0–5 °C. The solution was added dropwise to salicylic acid (1.38 g, 10 mmol), in 10% sodium hydroxide solution (25 mL) with stirring in an ice bath for a further 1 h. The produced monomer **I** was precipitated. The product was collected by filtration and washed with water (3×) and dried under vacuum at room temperature overnight. FTIR (KBr) cm^-1^: 3426 (-OH, *s*), 1573 (C=O, -COOH group, *s*), 682-834 cm^-1^ (benzene ring, *s*); ^1^H-NMR spectrum (DMSO-*d_6_*, ppm): δ 2.50 (singlet, -CH-CO-), 3.45 (broad singlet, -CH-N-), 7.74–8.25 (multiplet, H_arom_), 6.90–7.13 (multiplet, -OH), δ 10.20 (singlet, -COOH).

#### 3.4.2. Synthesis of 5-[4-(Hydroxyphenyl) azo] salicylic acid (HPAS, **II**)

The title monomer was synthesized as described for the synthesis of 3,3′-azobis(6-hydroxybenzoic acid) (**I**) using the following quantities of reagents: 5-aminosalicylic acid (1.53 g, 10 mmol), phenol (0.94 g, 10 mmol). FTIR spectrum (KBr) cm^-1^: 3423 (-OH, *s*), 1676 (C=O, -COOH group, *s*), 685–837 (benzene ring, *s*); ^1^H-NMR spectrum (DMSO-*d_6_*, ppm): δ 2.49 (singlet, -CH-CO-), 3.45 (broad singlet, -CH-N-), 7.63–8.25 (multiplet, H_arom_), 6.71–7.11 (multiplet, -OH), δ 10.40 (singlet, -COOH).

#### 3.4.3. Synthesis of 5-[1-Hydroxynaphthyl) azo] salicylic acid (HNAS, **III**)

The title monomer was synthesized as described with synthesis of 3,3′-azobis(6-hydroxy benzoic acid) (ABHB) (I) using the following quantities: 5-aminosalicylic acid (1.53 g, 10 mmol), α-naphthol (1.44 g, 10 mmol). FTIR (KBr) cm^-1^: 3407 (-OH, *s*), 1590 (C=O, -COOH group, *s*), 697–840 (benzene ring, *s*); ^1^H-NMR (DMSO-*d_6_*, ppm) δ 2.50 (singlet, -CH-CO-), 3.25 (broad singlet, -CH-N-), 7.77–8.29 (multiplet, H_arom_), 6.91–7.05 (multiplet, -OH), 10.20 (singlet, -COOH). 

### 3.5. Synthesis of Azo Polyurethanes

#### 3.5.1. Synthesis of Azo-polyurethane **IV** from Monomer **I** and 1,6-Hexamethylenediisocyanate

In a three-neck round-bottom flask, a solution of 3,3′-azobis(6-hydroxybenzoic acid) (ABHB, **I**) (3.01 g, 10 mmol), in *N*,*N*- dimethylformamide (DMF, 30 mL) was added dropwise to a solution of 1,6-hexamethylenediisocyanate (HDI, 1.68 g, 10 mmol), in dry DMF (20 mL), under a dry nitrogen atmosphere at room temperature. Then the reaction mixture was stirred at 80 °C for 8 h. The solution was poured into cold methanol to precipitate the polymer. The solid product **IV** was collected by filtration, washed with methanol, and dried under vacuum at room temperature. FTIR (KBr) cm^-1^: 3,332 (-OH, *s*), 2,931 (-CH_2_-, *s*), 1,618 (-CONH-, *s*), 1,573 (C=O, -COOH group, *s*), 1,376 (-CH (CH_3_)_2_-, *s*) and 639–830 cm^-1^ (benzene ring, *s*).

#### 3.5.2. Synthesis of Azo-polyurethane **V** from Monomer **II** and 1,6-Hexamethylenediisocyanate

The title polymer was synthesized as described for the synthesis of polymer **IV** using the following quantities: 5-[4-(hydroxyphenyl)azo]salicylic acid (HPAS, **II**, 2.57 g, 10 mmol), 1,6-hexamethylene-diisocyanate (HDI, 1.68 g, 10 mmol). FTIR (KBr) cm^-1^: 3331 (-OH, *s*), 2931 (-CH_2_-, *s*), 1623 cm^-1^ (-CONH-, *s*), 1572 (C=O, -COOH group, *s*) and 731–801 cm^-1^ (benzene ring, *s*).

#### 3.5.3. Synthesis of Azo-polyurethane **VI** from Monomer **III** and 1,6-Hexamethylenediisocyanate

The title polymer was synthesized as described with the synthesis of polymer **IV** using the following quantities: 2.5-[1-hydroxynaphthyl) azo] salicylic acid (HNAS, **III**, 3.08 g, 10 mmol), 1,6-hexamethylenediisocyanate (HDI, 1.68 g, 10 mmol). FTIR (KBr) cm^-1^: 3331 (-OH, *s*), 2859–2931 (-CH_2_-, *s*), 1622 (-CONH-, *s*), 1571 (C=O, -COOH group, *s*), and 631–729 cm^-1^ (benzene ring, *s*).

### 3.6. Determination of the Total 5-ASA Content

A sample of polymers **IV-VI** (10 mg) was suspended in PB (30 mL, pH 7.8). The mixture was heated at 60 °C, and the amount of 5-ASA released was determined using UV spectrophotometry at λ_max_ = 353 nm.

### 3.7. In vitro drug release

The release of 5-ASA was followed as a function of time using a UV spectrophotometer at λ_max_ = 353 nm. The procedure used was as follows: polymer (10 mg) was placed in aqueous buffer solution of pH = 7.8 and 1.5 at 37 °C (50 mL). At specific intervals, aliquots of the buffer (3 mL) were collected for analysis. Each experiment was carried out in triplicate.

## 4. Conclusions

Polyurethanes containing azo-5-ASA in the main chain were synthesized and characterized as polymeric biodegradable prodrug systems. The use of these prodrug systems can protect 5-ASA during its transfer to the colon and can be used for colon drug targeting. The rate of release depends on the pH of the medium and on the polymer structure. Generally, It was found that the rate of release of 5-ASA increased as the pH increased in alkaline medium (i.e. within the colon pH). Generally, as the number of carboxylic acid groups on monomeric units along the polymer chain increased, the hydrophilicity of the polymer increased, and the rate of hydrolysis increased and consequently, the amount of 5-ASA released increased. Moreover, the synthesized azo polymers showed good thermal stability and the onset decomposition temperature of all these polymers was found to be above 195 °C under nitrogen.

## Figures and Tables

**Table 1:** Elemental microanalysis of dihydroxyazobenzene derivatives based on 5-aminosalicylic acid **I**–**III** and their polymers **IV**–**VI**.

Product No.	C%		H%		N%	
	Calc.	Found	Calc.	Found	Calc.	Found
**I**	55.63	54.80	3.33	3.50	9.27	8.90
**II**	60.47	58.31	3.90	4.18	10.85	9.44
**III**	66.23	61.79	3.92	3.15	9.09	8.51
**IV**	55.96	56.02	4.17	4.91	10.88	9.80
**V**	59.65	57.91	4.71	9.30	12.27	11.60
**VI**	64.28	56.14	4.62	9.46	10.71	10.40

* Calc. = Calculated values.
